# Misdiagnosed Seizure-Like Activity in a Patient With Postural Orthostatic Tachycardia Syndrome: A Case Report

**DOI:** 10.7759/cureus.39565

**Published:** 2023-05-27

**Authors:** Sandra Safwat, Fady Safwat, Nivetha Sivanathan, Natashah Daka, Michael Sadek

**Affiliations:** 1 Internal Medicine, Washington University of Health and Science, San Pedro, BLZ; 2 Medicine, Washington University of Health and Science, San Pedro, BLZ; 3 Medicine, Richmond Gabriel University College of Medicine, Kingstown, VCT; 4 Medicine, Windsor University School of Medicine, Cayon, KNA; 5 Internal Medicine, Swedish Covenant Hospital, Chicago, USA

**Keywords:** postural orthostatic tachycardia syndrome (pots), pots treatment, neuropathic pots, hyperadrenergic pots, sinus tachycardia, recurrent syncope, potential pitfall for misdiagnosis, patient psychology, cardiology research, internal medicine (general medicine)

## Abstract

Postural orthostatic tachycardia is a syndrome characterized by an elevated heart rate in response to standing. This syndrome typically presents in late adolescence and early adulthood, with a higher percentage occurring in females. This syndrome is often seen following a viral infection, pregnancy, surgery, or intense psychological stress. This condition presents a wide range of symptoms that vary depending on its unclear etiology. We present the case of a 21-year-old woman with convulsions associated with postural orthostatic tachycardia syndrome after being misdiagnosed with a psychiatric condition for many years.

## Introduction

Postural orthostatic tachycardia syndrome (POTS) is one of the most common autonomic nervous system disorders (ANS) caused by exaggerated sinus tachycardia with upright posture [1]. Therefore, this condition can go undiagnosed for many years and is frequently misdiagnosed as anxiety disorder. These misdiagnoses likely occur because anxiety may be associated with tachycardia, palpitations and lightheadedness [1]. Furthermore, the following diagnostic criteria used to assist in identifying POTS include a sustained heart rate elevation of at least 30 bpm in adults and at least 40 bpm in teens 12-19 years of age from supine to standing position during a 10-min stand test or a tilt table test. Secondly, an absence of orthostatic hypotension and thirdly, symptoms of orthostatic intolerance must be present for at least six months [2]. A detailed medical history, physical examination with orthostatic vital signs or a brief tilt table test, and a resting 12-lead electrocardiogram are the most effective ways to identify POTS. The head-tilt test is a standardized method for evaluating an individual’s response to posture changes. As soon as baseline blood pressure and heart rate measurements have been taken, the patient is positioned on a standard tilt table and inclined to a 70-degree head-up position [3]. The heart rate and blood pressure are measured continuously or at least every 12 minutes. Often orthostatic tachycardia is detected by a similar threshold to the standing test (an increase of ≥30 bpm) [3]. It is, therefore, essential to interpret head-tilt testing cautiously and with respect to symptoms since “false-positive” orthostatic tachycardia can indeed be observed in the absence of typical POTS symptoms [4]. Additional diagnostic tests are required in some patients, depending on their clinical symptoms [4]. Orthostatic symptoms can include palpitation, chest pain or discomfort, lightheadedness, blurred vision, shortness of breath, headache, nausea, fatigue, and tremulousness [4]. Several factors can exacerbate these symptoms, including dehydration, heat exposure, prolonged recumbency, alcohol, and menstruation [4]. Given the rising awareness of the condition, recent epidemiology shows an increased incidence rate among younger individuals between the ages of 15-45, with a clear preeminence for the female population (≈80%) and primarily affects women of childbearing age [5].

This condition presents very broadly due to the vast amount of sub-etiologies that can co-exist simultaneously. The two significant hypotheses for the pathophysiology can be divided into neuropathic POTS, a form of parasympathetic denervation occurring in the lower extremities, forcing a burst of sympathetic outflow as a corrective effort. It can also be divided into hyperadrenergic POTS, an excessive outburst of norepinephrine when initiating an erect position. The etiology, like the symptoms, can be ambiguous. Specific literature on POTS suggests a possible combination of both or neither previously mentioned etiologies, prompting further investigation.

Physiologically, several mechanisms are in place to counteract the gravity-induced displacement of blood when an individual stands up. Without these mechanisms, a drop in systemic blood pressure is expected. Firstly, unstretched baroreceptors in the atrial and aortic walls would cause a decreased parasympathetic outflow, leading to increased heart rate. Correspondingly, a surge in sympathetic outflow would result in a compensatory increase in systemic vascular resistance. Additional redundancies like postural muscle contractions and upregulation of alpha-adrenergic receptors in the venous system work together to increase blood circulation back to the heart. Understanding defects or dysregulation in this system is vital to classifying the subtype of POTS and providing justification for the medications used to treat the condition.

## Case presentation

We present the case of a 21-year-old female admitted to the hospital for multiple episodes of syncope, followed by seizure-like activities. The patient has seizures associated with loss of consciousness for several seconds and subsequent tonic-clonic movements. During the episode, the patient would roll her eyes back and nod uncontrollably. There is no postictal state upon awakening. The patient endorses she has prodromal headaches when asked if there were any preceding events to the seizure-like activity. She denies any other symptoms before the episode, such as shortness of breath, palpitations, or visual disturbances. After an extensive history was taken, the patient endorsed that she was previously admitted multiple times for recurring symptoms of syncope and seizure-like activity. Significant history includes a previous exacerbating event, where she had been standing for a prolonged period and subsequently developed syncope. The parents also note a recurring theme of worsening symptoms 10 minutes postprandially. There are no alleviating factors.

Past medical history is significant for depression, anxiety, anemia, and vitamin D deficiency. Her family history includes a paternal uncle with arrhythmia who died from a heart attack at 48. The patient has allergies to cephalexin. Clinical examination was significant for two events of syncope associated with convulsions. A complete neurological workup, including a brain MRI and CT scan, was performed and showed to be unremarkable. An EEG was performed to assess the seizure-like activity. The EEG showed no abnormalities with no abnormal slowing, epileptiform discharges, or seizures recorded. ECG was also performed and showed no abnormalities. A diagnosis of conversion disorder was presumed due to the rare seizure-like activity in POTS. The patient was diagnosed with a head-tilt test which showed to be positive. She was elevated to a 75-degree angle and her heart rate went up to 133 within 1 minute, from 73 at rest (Table 1).

**Table 1:** Tilt-table test results with body elevation at 75 degrees for 16 minutes, after which the study was aborted at 16 minutes secondary to intolerance BP: blood pressure, HR: heart rate, bpm: beats per minute, mmHg: millimeters of mercury

	Supine position	1 minute	5 minutes	16 minutes
BP (mmHg)	113/63	143/77	136/72	139/75
HR (bpm)	73	133	142	165

The patient was discharged home with a prescription for fludrocortisone 0.1 mg orally (PO) daily and famotidine 10 mg twice daily as needed for reflux. She was counseled on the appropriate lifestyle modifications to minimize her symptoms. She was also referred to a cardiologist and neurologist. No complications or new clinical findings were identified as of the time of writing.

## Discussion

POTS is a relatively uncommon condition, with a prevalence of 0.2% in the general population [5]. Physicians commonly miss this syndrome due to the wide variance of case presentations. This article showcases a rare presentation of POTS to educate and inform the general population. This article, moreover, exhibits how misdiagnosing patients can regularly lead to consequent conditions, such as anxiety. Highlighting various ways POTS may present can substantially decrease the percentage of physicians misdiagnosing POTS. A case report study by Kesserwani (2020) highlights how commonly mistaken POTS is for anxiety disorder due to its hyperadrenergic side effects [6]. We can appreciate how, similarly, in this case, the patient has suffered from anxiety disorder since the age of 17 and was only diagnosed at 21 with POTS. This study highlights the significance of prompt history-taking in patients to make a correct diagnosis. To further show the delay in diagnosis, a study by Shaw et al. (2019) highlights the statistics related to the misdiagnosis of POTS in the general population and how there is often a delay in the diagnosis [7]. Three‐quarters (n = 3421; 75%) of patients report that a physician misdiagnosed their POTS symptoms before being diagnosed with POTS. In addition, a high percentage (77%) of participants reported being told they were suffering from a psychiatric or psychological problem before their POTS diagnosis [7]. However, in contrast, only 1247 (28%) respondents reported suffering from a psychiatric or psychological problem. Despite this improvement, the average diagnostic delay since 2009 is still over 4.7 ± 6.9 years [median 23 (IQR 6-69) months] [7]. We see this prevail clinically with our patient as the doctor visits after the diagnosis of POTS have displayed a clinically less anxious individual, indicating that years of misdiagnosis coupled with the uncertainty of her condition led to daily life distress. In another case report by Sidhu et al., a 33-year-old female with recurrent syncope and palpitations was told by physicians that the syncope was related to stress for many years [8].

Another unique finding in this case report was the tonic-clonic convulsions witnessed in our patient despite the negative findings during a complete neurological workup. In a case study conducted by Gadze et al. (2018), they describe the case of a 29-year-old female patient with pharmacoresistant epilepsy from the age of 9 that was confirmed with magnetic resonance imaging (MRI) [9]. The symptoms of their case report presented as focal impaired awareness seizures of motor onset with gestural automatisms and motor dysphasia, followed by a sense of fear and panic attacks, with short-term postictal confusion, corresponding to left temporal lobe seizures [9]. The case also highlights the disappearance of the symptoms of POTS and the seizures following the use of vagal nerve stimulation (VNS), which can indicate that both symptoms originate from the exact etiology rather than two distinct disorders. While 40 percent of patients with POTS may present with syncope, convulsion similar to that which our patient experiences is a rare finding, even more so with the addition of the negative MRI and CT scan findings. Another interesting case report by Kim et al. (2018) describes the case of a young woman with recently diagnosed POTS and narcolepsy following viral illness-induced lesions to the thalamus and amygdala [10]. The POTS was diagnosed with tilt table testing following a positive brain MRI showing hyper-intensive lesions in the thalamus and amygdala [10]. These reports help highlight two fascinating points: POTS may be a central nervous system disorder versus a peripheral nervous one. The second is a crucial need for further workup for orthostatic intolerance beyond a neurological workup, as seen in our patient, who did not have a typical etiology and an entirely negative neurological assessment. Due to the negative neurological workup of our patient, the diagnosis of conversion disorder or psychogenic seizures was strongly considered in this patient. In addition, the limited literature to support the finding of convulsions associated with POTS prompted the inclusion of the diagnosis in our differential.

## Conclusions

​It is paramount that more medical practitioners be aware that the diagnostic criteria are a clinical entity that is now widely recognized and that there is an agreement on its diagnostic standards. Due to the wide range of sub-etiologies that can co-exist simultaneously, as well as the variety of symptoms and non-specificity of the presentation of this disorder, POTS is frequently misdiagnosed. This article aims to inform and educate the general public by highlighting the diagnostic criteria for postural orthostatic tachycardia syndrome, its clinical features, pathophysiology, and the various manifestations. More research and literature are required to understand the pathophysiology better.
